# A Correction Approach for the Inclined Array of Hydrophones in Synthetic Aperture Sonar

**DOI:** 10.3390/s18072000

**Published:** 2018-06-22

**Authors:** Haoran Wu, Jinsong Tang, Heping Zhong

**Affiliations:** Naval Institute of Underwater Acoustic Technology, Naval University of Engineering, Wuhan 430033, China; wuhaoran_wh@163.com (H.W.); zheping525@sohu.com (H.Z.)

**Keywords:** synthetic aperture sonar, the array of hydrophones, yaw, pitch

## Abstract

A correction approach for the inclined array of hydrophones is proposed to prevent decline of the image quality in SAS. In this approach, the 2-way exact acoustic propagation path of the inclined array is transformed into the sum of a single root term and an offset term, where the single root term is the 2-way ideal propagation path and the offset term contains all errors cause by the inclined array. The correction for the offset term is separated into two steps: phase correction and delay correction. The phase correction is performed on the echo signal of each receiving hydrophone in the 2-D time domain by a phase multiplication and the delay correction is performed on the echo signal of each receiving hydrophone in the range frequency domain by a phase multiplication with a linear function of range frequency at the reference range. Finally, the effectiveness of the proposed approach is examined by the simulation experiments.

## 1. Introduction

Synthetic aperture sonar (SAS) [1] is emerging as a remote sensing technology that can provide centimeter resolution over a hundreds-of-meters range on the seafloor [2]. Because of the low speed of sound in water, a SAS requires an array of receiving hydrophones to obtain a useful mapping rate [3,4,5,6]. However, the array is inclined by the turbulence, waves and ocean current [7] in the changeable underwater environment, and it is not parallel to the motion direction. In this case, the positions of the receiving hydrophones are deviated from the ideal straight trajectory, so it will cause errors in the acoustic propagation path [8], by which the image quality will be degraded. Normally, the errors are expected to be less than one eighth of the carrier wavelength [9]. This is a very high requirement for a SAS in the uncertain underwater environment. Thus, some corrections for the errors must be made to prevent the decline of the image quality.

Generally, there are three angles utilized to describe the array of SAS, which are referred to as yaw, pitch and roll and are defined in [10]. Because the typical vertical beam-width is 20° to 50° and the size of array in the vertical direction is negligible compared with the distance between the array and the targets [11], the array can be viewed as a line array, where the phase centers of transmitter and receiving hydrophones are located on the center axis of the array. Considering that roll is the angle that the array rotates around its own center axis, it will not change the position of phase center and does not bring the acoustic propagation path error. Thus, roll is not a significant defocusing factor, and only yaw and pitch need to be taken into account. At present, most of work reported is based on the assumption that only yaw has an impact on the image quality [8,12,13,14,15,16,17,18], and there is little research reported that yaw and pitch both have an impact on the image quality. The approaches of the yaw-only correction can be classified into three categories: time-domain beamforming method [19,20,21], preprocessing correction method [12], and imaging autofocus method [3]. The time-domain beamforming method can accurately correct arbitrarily yaw angle, but it is computationally expensive. The preprocessing correction method is usually implemented separately as a preprocessing step before using the more efficient block reconstruction algorithms [22], such as the range Doppler algorithm (RDA), chirp scaling algorithm (CSA), and range migration algorithm (RMA), and the correction is performed in the preprocessing step. The imaging autofocus method supposes that the blurring in the reconstructed image is all in the along-track direction. This means that any uncorrected yaw errors need to be much smaller than the across resolution. In cases where the yaw and pitch are both taken into account, Hansen [2] points out that a small yaw or pitch error will cause periodic errors in the synthetic aperture, where the periodicity is constructed by the array, but a corresponding correction method is not proposed, and Huang [23] proposes a correction method for yaw and pitch, but his method can only be used together with the inverse scaled Fourier transformation algorithm. 

In this paper, we propose a correction approach for the inclined array of hydrophones in SAS. Because of building on the 3-D geometric model of the multi-hydrophone SAS, the analytic expression of the 2-way exact acoustic propagation path with yaw and pitch can be obtained. Considering the complexity of this expression, we utilize some approximations to simplify it, and get a 2-way approximated acoustic propagation path that contains a single root term and an offset term, where the single root term is viewed as an 2-way ideal propagation path of the acoustic signal that is transmitted and collected on the ideal trajectory, and the offset term contains all errors that need to be corrected. Thus, the correction can be performed only by compensating the offset term in echo signal, which contains phase correction and delay correction. Because the offset term is independent of the azimuth time, the phase correction can be performed on echo signals of each hydrophone in the 2-D time domain by a phase multiplication. Then, considering that the offset term is weakly dependence of the range, its delay can be viewed as the same as the delay along the range [24], which can be replaced by the delay at the reference range, which is generally the middle distance of image scene. Then, according to the Fourier transform shifting properties, the operation that shifts the echo signal by a constant delay in the time domain can be performed on the echo signal in the range frequency domain by a phase multiplication with a linear function of range frequency at the reference range. Finally, the effectiveness of our approach was examined by the simulation experiment.

The rest of the paper is organized as follows. The proposed approach is developed in Section 2. Section 3 evaluates the performance of the proposed approach by the simulation experiment. Some conclusions are given in Section 4.

## 2. The Development of Correction Approach

### 2.1. The Exact Acoustic Propagation Path

**Figure 1:**
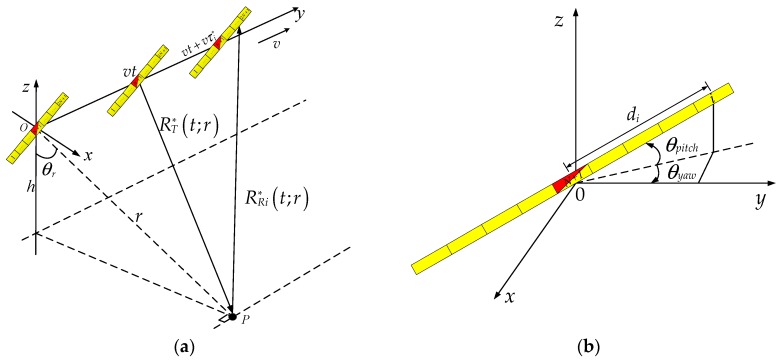
Geometric model of the multiple hydrophone SAS. (**a**) The acoustic propagation path; (**b**) The inclined array of hydrophones.

As shown in Figure 1a, SAS motion direction is the *y*-axis, and the *x*-axis is horizontal, perpendicular to the *y*-axis, and the *z*-axis is pointed upward. The array of SAS contains *2N + 1* receiving hydrophones and one transmitter, where the (*N + 1*)th element is located at the middle of the array and is shared by the transmitter and the (*N + 1*)th receiving hydrophone. *d_i_* is the baseline between the transmitter and the *i*th receiving hydrophone, which is equal to [i−(N+1)]Δd (Δd is the interval between the hydrophones). The rotation center of the array is the transmitter, and *θ_pitch_* and *θ_yaw_* are pitch and yaw, as shown in Figure 1b, and a positive pitch and yaw are defined as a clockwise rotational movement. *v* is the speed of SAS, *t* is the azimuth time, *h* is the height of the array from seafloor, and zero azimuth time (t=0) is defined as the moment that the transmitter cross the origin of coordinate. There is a point target that is located at (*r*sin*θ_r_*, *0*, *h*), where *r* is the shortest range between the transmitter and the point target, and *θ_r_* is the depression angle between *r* and the *z*-axis. 

The inclined array moves along the direction of *y*-axis. When the transmitter is located at the position *vt* in *y*-axis, the 1-way propagation path of the transmitter to *P* is

(1) $$R_{T}{}^{*}\left(t;r\right)=\sqrt{r^{2}+v^{2}t^{2}}$$

Because of the low speed of sound in water, the moving distance of SAS between transmitting and receiving signal is not negligible and should be taken into account. This mode is referred to as ‘non-stop-hop-stop’ [25,26]. Supposing the delay between transmitting and receiving signal is $\tau_{i}^{*}$, the moving distance of SAS is $v\tau_{i}^{*}$ and the *i*th receiving hydrophone is located at the position $\left(d_{i}\cos\theta_{pitch}\sin\theta_{yaw},vt+v\tau_{i}^{*}+d_{i}\cos\theta_{pitch}\cos\theta_{yaw},d_{i}\sin\theta_{pitch}\right)$ when it collects the scattered signal of the point *P*. Thus, the 1-way propagation path of *P* to the *i*th receiving hydrophone is

(2) $$R_{Ri}{}^{*}\left(t;r\right)=\sqrt{r^{2}+Q+{\left(vt+v\tau_{i}^{*}+d_{i}\cos\theta_{pitch}\cos\theta_{yaw}\right)}^{2}}$$

where *Q* is represented as

(3) $$Q=d_{i}^{2}\cos^{2}\theta_{pitch}\sin^{2}\theta_{yaw}+d_{i}^{2}\sin^{2}\theta_{pitch}+2hd_{i}\sin\theta_{yaw}-2rd_{i}\cos\theta_{pitch}\sin\theta_{yaw}\sin\theta_{r}$$

According to Equations (1) and (2), the 2-way exact propagation path is obtained by

(4) $$R_{i}{}^{*}\left(t;r\right)=R_{T}{}^{*}\left(t;r\right)+R_{Ri}{}^{*}\left(t;r\right)$$

Because the acoustic signal has propagated for $\tau_{i}^{*}$ when it is collected by the *i*th hydrophone, the 2-way exact propagation path is also written by

(5) $$R_{i}{}^{*}\left(t;r\right)=c\tau_{i}^{*}$$

where *c* represents the speed of the underwater sound. Combining Equations (4) and (5), the solution for $\tau_{i}^{*}$ is

(6) $$\tau_{i}^{*}=\frac{B+\sqrt{B^{2}+AC}}{A}$$

where *A*, *B*, and *C* are respectively represented as follows:

(7) $$A=c^{2}-v^{2}$$

(8) $$B=vd_{i}\cos\theta_{yaw}\cos\theta_{pitch}+v^{2}t+c\sqrt{v^{2}t^{2}+r^{2}}$$

(9) $$C=2vtd_{i}\cos\theta_{yaw}\cos\theta_{pitch}+d_{i}^{2}\cos^{2}\theta_{yaw}\cos^{2}\theta_{pitch}+Q$$

### 2.2. The Approximated Acoustic Propagation Path

**Figure 2:**
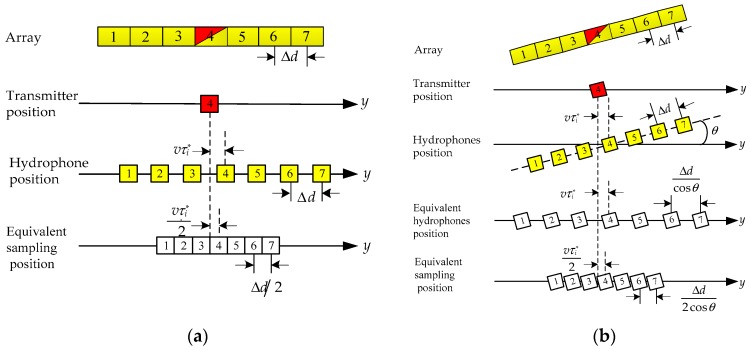
The position of the transmitter and the hydrophones, and the azimuth position of the phase center, when the sampling point is uniformly distributed (7 hydrophones in this example). (**a**) Side-looking array; (**b**) Inclined array.

To make use of the existing monostatic synthetic aperture imaging algorithm [22], such as RDA, CSA and RMA, etc., it is necessary to transform the multiple hydrophone signals into a single hydrophone signal by the preprocessing step [27]. As a comparison, Figure 2a shows how the multiple hydrophone signals are transformed in the case without inclination. In Figure 2a, the equivalent sampling position of a single hydrophone is the midpoint between the position of the transmitted signal and the position of the collected signal. 

In the case for the inclined array, the equivalent sampling position is different from that in Figure 2a because of the array deviated from the *y*-axis. As shown in Figure 2b, the receiving hydrophones in the array need to be equivalent to the *y*-axis before getting the equivalent sampling position. According to geometric model in Figure 1b, the angle of the array deviated from the *y*-axis *θ* is obtained by

(10) $$\cos\theta =\cos\theta_{yaw}\cos\theta_{pitch}$$

In Figure 2b, the interval length between equivalent hydrophones is $\frac{\Delta d}{\cos\theta }$. To ensure uniform sampling in the *y*-axis direction, the pulse repetition frequency (PRF) should be adjusted from $\frac{2v}{\left(2N+1\right)\Delta d}$ to $\frac{2v\cos\theta }{\left(2N+1\right)\Delta d}$ Because *θ* is usually a small angle, $\frac{2v\cos\theta }{\left(2N+1\right)\Delta d}$ can be approximated into $\frac{2v}{\left(2N+1\right)\Delta d}$ [24] and the interval between equivalent sampling positions is approximated into $\frac{\Delta d}{2}$ [8]. Thus, the 2-way propagation path at the equivalent sampling point is

(11) $$R_{i}^{\prime }\left(t;r\right)=2\sqrt{r^{2}+{\left(vt+v\frac{\tau_{i}^{*}}{2}+\frac{\Delta d}{2}\right)}^{2}}$$

where the square root term is a 2-way ideal propagation path without any error. To make $R_{i}^{\prime }\left(t;r\right)$ closer to the 2-way exact acoustic propagation path, an offset term is necessary. Combining Equations (4) and (11), the offset term is obtained by

(12) $$\begin{array}{cc} \Delta R\left(r;d_{i}\right) & =R_{i}{}^{*}\left(t;r\right)-R_{i}^{\prime }\left(t;r\right)\\ & =\sqrt{r^{2}+v^{2}t^{2}}+\sqrt{r^{2}+Q+{\left(vt+v\tau_{i}^{*}+d_{i}\cos\theta \right)}^{2}}-2\sqrt{r^{2}+{\left(vt+v\frac{\tau_{i}^{*}}{2}+\frac{d_{i}}{2}\right)}^{2}} \end{array}$$

where $\tau_{i}^{*}$ is the delay of the 2-way exact propagation path. It is necessary to simplify $\tau_{i}^{*}$ before $\Delta R\left(r;d_{i}\right)$ is simplified further. Since $\tau_{i}^{*}$ is weak azimuth dependence and strong range dependence, it can be replaced by 2r/c [27]. Correspondingly, Equation (12) is rewritten as

(13) $$\Delta R\left(r;d_{i}\right)=\sqrt{r^{2}+v^{2}t^{2}}+\sqrt{r^{2}+Q+{\left(vt+v\frac{2r}{c}+d_{i}\cos\theta \right)}^{2}}-2\sqrt{r^{2}+{\left(vt+v\frac{r}{c}+\frac{d_{i}}{2}\right)}^{2}}$$

The following equation is used to simplify $\Delta R\left(r;d_{i}\right)$.

(14) $$\sqrt{1+\varsigma }\approx 1+\frac{\varsigma }{2}-\frac{\varsigma^{2}}{8}$$

Substituting Equation (14) into Equation (13), $\Delta R\left(r;d_{i}\right)$ is rewritten as

(15) $$\begin{array}{cc} \Delta R\left(r;d_{i}\right) & =r\left(1+\frac{v^{2}t^{2}}{2r^{2}}-\frac{v^{4}t^{4}}{8r^{4}}\right)\\ & +r\left\{1+\frac{1}{2r^{2}}\left[Q+{\left(vt+v\frac{2r}{c}+d_{i}\cos\theta \right)}^{2}\right]-\frac{1}{8r^{4}}{\left[Q+{\left(vt+v\frac{2r}{c}+d_{i}\cos\theta \right)}^{2}\right]}^{2}\right\}\\ & -2r\left[1+\frac{1}{2r^{2}}{\left(vt+v\frac{r}{c}+\frac{d_{i}}{2}\right)}^{2}-\frac{1}{8r^{4}}{\left(vt+v\frac{r}{c}+\frac{d_{i}}{2}\right)}^{4}\right] \end{array}$$

Next, $\Delta R\left(r;d_{i}\right)$ in the narrow beam system can be replaced by the $\Delta R\left(r;d_{i}\right)$ at the beam center and the terms of $1/r^{3}$ are ignored. Thus, $\Delta R\left(r;d_{i}\right)$ is obtained by

(16) $$\Delta R\left(r;d_{i}\right)=\frac{Q}{2r}+\frac{1}{2r}{\left(v\frac{2r}{c}+d_{i}\cos\theta \right)}^{2}-\frac{1}{4r}{\left(v\frac{2r}{c}+d_{i}\right)}^{2}$$

Then, Equation (16) is expanded as

(17) $$\Delta R\left(r;d_{i}\right)=\frac{1}{4r}{\left(v\frac{2r}{c}+d_{i}\right)}^{2}+\frac{Q}{2r}+\frac{d_{i}^{2}}{2r}\left(\cos^{2}\theta -1\right)+\frac{2vd_{i}}{c}(\cos\theta -1)$$

Substituting Equations (3) and (10) into Equation (4), $\Delta R\left(r;d_{i}\right)$ is simplified as

(18) $$\Delta R\left(r;d_{i}\right)=\frac{1}{4r}{\left(v\frac{2r}{c}+d_{i}\right)}^{2}-d_{i}\cos\theta_{pitch}\sin\theta_{yaw}\sin\theta_{r}+\frac{hd_{i}\sin\theta_{yaw}}{r}$$

where the first term is the relative range offset [28] caused by the configuration of multiple hydrophones and the non-stop-hop-stop mode, the second term is the path errors cause by pitch and yaw, and the third term is the path errors cause by only yaw.

Combining $R_{i}^{\prime }\left(t;r\right)$ and $\Delta R\left(r;d_{i}\right)$, the 2-way approximated acoustic propagation path with errors is given by

(19) $$R_{i}\left(t;r\right)=2\sqrt{r^{2}+{\left(vt+v\frac{r}{c}+\frac{d_{i}}{2}\right)}^{2}}+\Delta R\left(r;d_{i}\right)$$

where the first term is a single root term that is a 2-way ideal propagation path obtained at the equivalent sampling position, and the second term is the offset term containing all errors that need to be corrected.

### 2.3. The Echo Signal Model

After demodulation, the echo signal collected by the *i*th hydrophone can be written by

(20) $$s_{i}(t,\tau ;r)=w_{r}\left(\tau -\frac{R_{i}\left(t;r\right)}{c}\right)\omega_{az}\left(t\right)\exp\left\{-j\frac{2\pi f_{0}R_{i}\left(t;r\right)}{c}\right\}\exp\left\{j\pi K_{r}{\left(\tau -\frac{R_{i}\left(t;r\right)}{c}\right)}^{2}\right\}$$

where *w*(·) represents the envelope of the transmitted signal, $\omega_{az}\left(\cdot \right)$ represents the beam pattern of transponder and one hydrophone, *τ* is the range time, *K_r_* is the FM rate of the transmitted signal, and *f*_0_ is the center frequency of the transmitted signal.

### 2.4. The Correction Process

**Figure 3:**
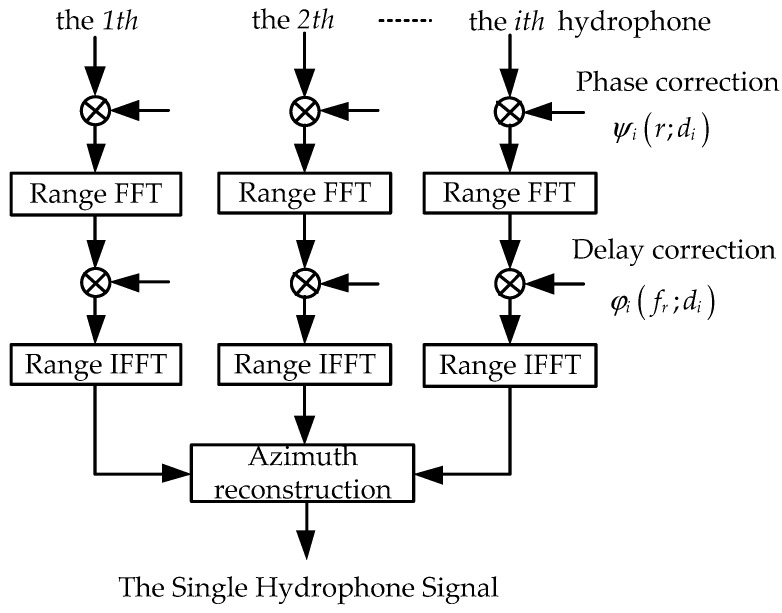
Flow chart of correction.

The correction removing $\Delta R\left(r;d_{i}\right)$ from the echo signal is separated into five steps, as shown in Figure 3. 

The first step is the phase correction. It can be seen from Equation (18) that $\Delta R\left(r;d_{i}\right)$ is related to the range *r* and the baseline *d_i_*, so the phase correction is performed on the echo signal of each hydrophone in the 2-D time domain by a phase multiplication at the every range bin. Combining Equations (19) and (20), the factor for this phase multiplication is

(21) $$\psi_{i}\left(r;d_{i}\right)=\exp\left\{j\frac{2\pi f_{0}}{c}\Delta R\left(r;d_{i}\right)\right\}$$

Then, after multiplying Equation (20) with Equation (21), the signal corrected phase is

(22) $$s_{i}^{\prime }(t,\tau ;r)=w_{r}\left\{\tau -\frac{R_{i}^{\prime }\left(t;r\right)+\Delta R\left(r;d_{i}\right)}{c}\right\}\omega_{az}\left(t\right)\exp\left\{-j\frac{2\pi f_{0}R_{i}^{\prime }\left(t;r\right)}{c}\right\}\exp\left\{j\pi K_{r}{\left[\tau -\frac{R_{i}^{\prime }\left(t;r\right)+\Delta R\left(r;d_{i}\right)}{c}\right]}^{2}\right\}$$

The second step is the range Fourier transformation, and $s_{i}^{\prime }\left(t,\tau ;r\right)$ is transformed to the range frequency domain. Here, we utilize the principle of stationary phase (POSP) [22] to perform the range Fourier transform. Correspondingly, the signal in the range frequency domain can be written by

(23) $$\begin{array}{cc} S_{i}^{\prime }(t,f_{r};r) & =\int_{-\infty }^{\infty }s_{i}^{\prime }(t,\tau ;r)\exp\left(-j2\pi f_{r}\tau \right)d\tau \\ & =W_{r}\left(f_{r}\right)\omega_{az}\left(t\right)\exp\left\{-j\frac{2\pi f_{0}R_{i}^{\prime }\left(t;r\right)}{c}\right\}\exp\left\{-j\frac{\pi f_{r}^{2}}{K_{r}}\right\}\exp\left\{-j\frac{2\pi f_{r}}{c}\left[R_{i}^{\prime }\left(t;r\right)+\Delta R\left(r;d_{i}\right)\right]\right\} \end{array}$$

where $W_{r}\left(\cdot \right)$ represents the spectral envelope of the transmitted signal and $f_{r}$ is the range frequency.

The third step is the delay correction. Because $\Delta R\left(r;d_{i}\right)$ is weakly dependent on the range [24], it can be replaced by the delay at the reference range *r_ref_*. Then, the correction for the constant delay is performed on the echo signal of each hydrophone in the range frequency domain by a phase multiplication. Combining Equations (19) and (20), the factor for this phase multiplication is

(24) $$\varphi_{i}\left(f_{r};d_{i}\right)=\exp\left\{j2\pi \frac{\Delta R\left(r_{ref};d_{i}\right)}{c}f_{r}\right\}$$

Then, after multiplying Equation (23) with Equation (24), the signal corrected delay is obtained by

(25) $$S_{i}^{\prime \prime }(t,f_{r};r)\approx W_{r}\left(f_{r}\right)\omega_{az}\left(t\right)\exp\left\{-j\frac{2\pi \left(f_{0}+f_{r}\right)R_{i}^{\prime }\left(t;r\right)}{c}\right\}\exp\left\{-j\frac{\pi f_{r}^{2}}{K_{r}}\right\}$$

The fourth step is the range inverse Fourier transform. Here, we again utilize POSP to perform the range inverse Fourier transform on $S_{i}^{\prime \prime }\left(t,f_{r};r\right)$. Then the signal in the 2-D time domain is

(26) $$\begin{array}{cc} s_{i}^{\prime \prime }(t,\tau ;r) & =\int_{-\infty }^{\infty }S_{i}^{\prime }(t,f_{r};r)\exp\left(j2\pi f_{r}\tau \right)df_{r}\\ & =w_{r}\left\{\tau -\frac{R_{i}^{\prime }\left(t;r\right)}{c}\right\}\omega_{az}\left(t\right)\exp\left\{-j\frac{2\pi f_{0}R_{i}^{\prime }\left(t;r\right)}{c}\right\}\exp\left\{j\pi K_{r}{\left[\tau -\frac{R_{i}^{\prime }\left(t;r\right)}{c}\right]}^{2}\right\} \end{array}$$

The fifth step is the azimuth reconstruction. After the phase and delay correction, the multiple hydrophone signals $s_{i}^{\prime \prime }\left(t,\tau ;r\right)$ can be viewed as the signal collected by a single hydrophone at the equivalent sampling position in Figure 2b. Thus, they can be transformed into a single hydrophone signal by the method that the signals are arranged in a sequence of hydrophones and pulses. Then, the reconstructed signal is

(27) $$s(t,\tau ;r)=w_{r}\left\{\tau -\frac{R\left(t;r\right)}{c}\right\}\omega_{az}\left(t\right)\exp\left\{-j\frac{2\pi f_{0}R\left(t;r\right)}{c}\right\}\exp\left\{j\pi K_{r}{\left[\tau -\frac{R\left(t;r\right)}{c}\right]}^{2}\right\}$$

(28) $$R\left(t;r\right)=2\sqrt{r^{2}+{\left(vt+v\frac{r}{c}\right)}^{2}}$$

where R(t;r)  can be viewed as a 2-way ideal propagation path of the acoustic signal that is transmitted and collected at the same position vt+vr/c. Thus, the existing imaging algorithms of the monostatic SAS can be modified to perform the image reconstruction on s(t,τ;r)  [29].

## 3. Results

To verify the effectiveness of the approach proposed in this paper, the computer simulation experiment is carried out in this section.

### 3.1. Simulation Parameters

The system parameters are given in Table 1, which are similar parameters to a real multiple hydrophones SAS.

### 3.2. Approximate Errors

**Figure 4:**
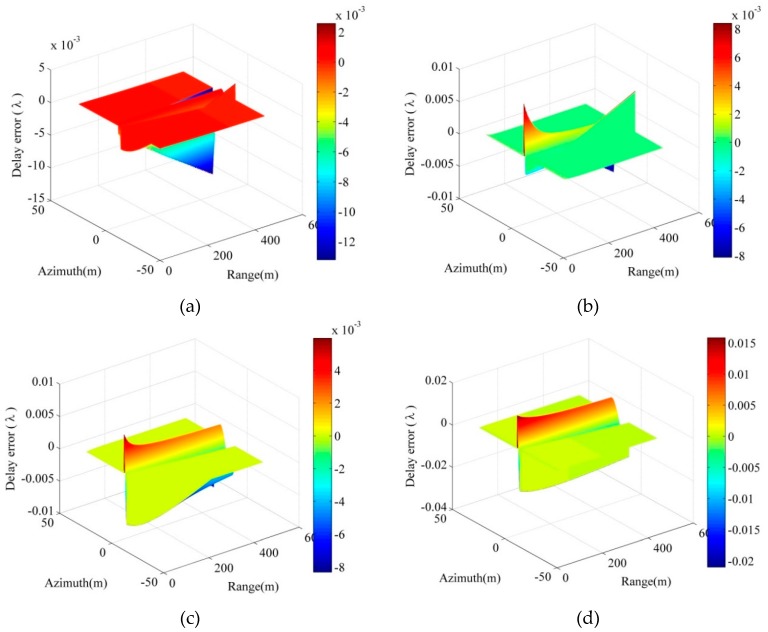
Delay errors. (**a**) 2° yaw angle and 0° pitch angle; (**b**) 0° yaw angle and 2° pitch angle; (**c**) 2° yaw angle and 2° pitch angle; (**d**) 3° yaw angle and 3° pitch angle.

As previously described, some approximations are utilized in the 2-way exact acoustic propagation path. Here, we evaluated the size of the delay error cause by the approximation under different yaw angles and pitch angles. The system parameters utilized are shown in Table 1, and the delay errors of the approximated acoustic propagation path $R_{i}\left(t;r\right)$ are shown in Figure 4.

It is observed from Figure 4 that yaw angle and pitch angle both have an influence on the size of the delay error. Moreover, we can see in Figure 4a,b that the delay errors cause by yaw angle for the far target are greater than the near target, and the delay errors caused by pitch angle for the beam edge are far greater than for the beam center. In addition, it is found that the array with greater yaw angle and greater pitch angle has a greater delay error by comparing Figure 4c,d. Considering that the size of the delay error should be less than 0.125λ (λ represents the wavelength of the signal carrier) [9], we measured the maximum delay error in Figure 4. The measured results of Figure 4a–d are 0.0158λ, 0.0165λ, 0.0143λ and 0.0368λ, respectively, which are far lower than 0.125λ. Therefore, it can be concluded that the size of the delay error for the approximated acoustic propagation path has no effect on the imaging results in the case of the small yaw angles and pitch angles. 

### 3.3. Imaging Results

**Figure 5:**
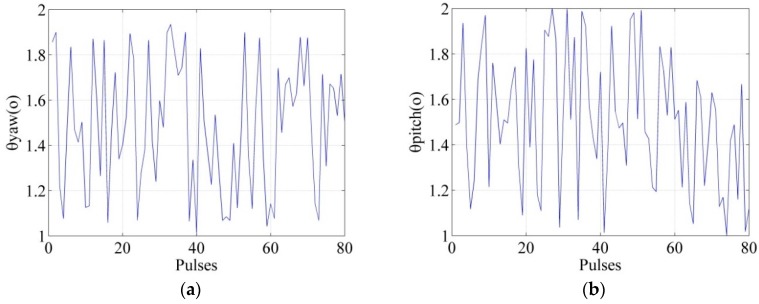
Random yaw angle and pitch angle. (**a**) The yaw angle; (**b**) The pitch angle.

In this section, the performance of the proposed approach in this paper is evaluated by comparing the imaging quality before and after compensation. The system parameters are shown in Table 1, and the 2-way exact acoustic propagation path of the targets are given by Equation (5). The scene illuminated by sonar has five idea point targets, which are assumed to be located at the position P_1_(297 m, −3 m), P_2_(303 m, −3 m), P_3_(303 m, 3 m), P_4_(297 m, 3 m), and P_5_(300 m, 0 m), respectively. In the simulation, we consider that the yaw angle and the pitch angle are random between 1° and 2° as shown in Figure 5, and the imaging algorithm utilized is the nonlinear chirp scaling algorithm [24].

Considering that most of work reported is based on the assumption that only yaw angle has an impact on the image quality [8,12,13,14,15,16,17,18], we perform the correction in three cases: only pitch angle correction, only yaw angle correction, and both pitch angle and yaw angle correction. It is important to note that the proposed approach in this paper is the case of both pitch angle and yaw angle correction.

**Figure 6:**
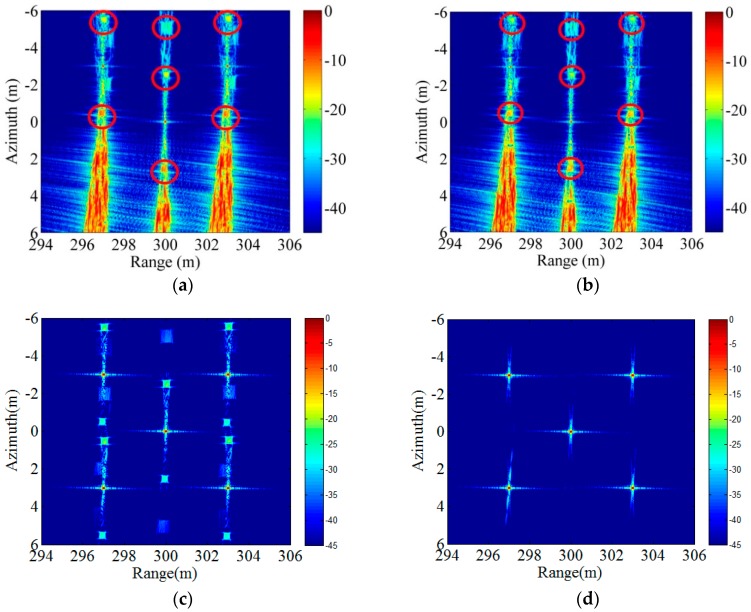
The imaging results. (**a**) No correction; (**b**) Only pitch angle correction; (**c**) Only yaw angle correction; (**d**) Both pitch angle and yaw angle correction.

It can be seen that the pitch angle correction reduces the energy of some false targets by comparing the red circle part in Figure 6a,b. Moreover, by comparing Figure 6a and Figure 6c, it is found that the targets have been well focused but there are still some false targets with strong energy when only yaw angle correction. These compared results show that the yaw angle correction has a significant impact on the imaging quality, and the pitch angle correction can further improve the imaging quality. In addition, it is found in Figure 6d that all targets are well focused and there is no false target after the array is corrected by the proposed approach in this paper. In order to compare the imaging results in more detail, the azimuth and range slice of the point P_1_ and P_5_ are shown in Figure 6. Then their impulse response width (IRW), peak sidelobe ratio (PSLR) and sidelobe ratio (ISLR) are measured, and the results are shown in Table 2.

**Figure 7:**
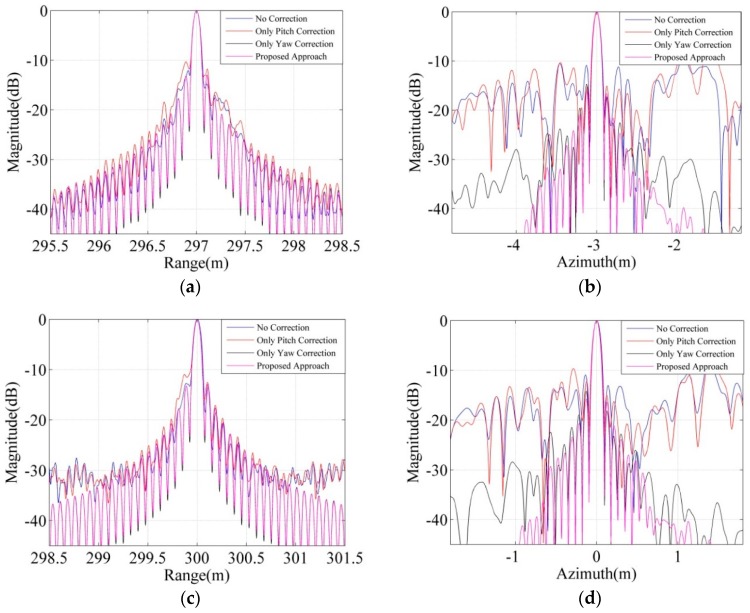
The magnitude of the azimuth slice and range slice for the point P_1_ and P_5_ in the case of the constant yaw angle and pitch angle. (**a**) The range slice of the point P_1_; (**b**) The azimuth slice of the point P_1_; (**c**) The range slice of the point P_5_; (**d**) The azimuth slice of the point P_5_.

Figure 7a–d shows the magnitude of the range slice and of azimuth slice for the point P_1_ and for the point P_5_ in the different correction cases, respectively. The results of Figure 7a,c and Table 2 show that yaw angle and pitch angle have little effect on the range image. Theoretically, the range image is obtained by the match filter technology; thus, the error of acoustic propagation path has less effect on the range image than the azimuth image. In Figure 7b,d, there are many false targets with strong energy if no correction is performed. It is obviously found that the azimuth images become very pretty after the proposed approach is used. Therefore, the results in Figure 6 and Figure 7 and Table 2 can get some conclusions that the yaw angle correction mainly affects the focusing performance for the target and most of the energy of false target, the pitch angle correction mainly affects a part of the energy of the false targets, and the approach proposed by this paper is effective. 

## 4. Conclusions

In this paper, we have proposed a correction approach for the inclined array of the hydrophones to prevent decline of the resulting image quality in SAS. Our approach can correct yaw angle and pitch angle simultaneously, while most other approaches only consider the yaw angle. In our simulation experiments, the important of the pitch angle correction is proven. Finally, the effectiveness of the proposed approach is examined by the simulation experiments. 

## Figures and Tables

**Table 1:** System parameters.

Carrier Frequency/kHz	70	Pulse Width/ms	20	PRF/Hz	2.3436
Bandwidth/kHz	10	Speed/m/s	3	Hydrophone/m	0.08
Range Sample Rate/kHz	20	Transmitter/m	0.16	Hydrophone Number	32

**Table 2:** The parameters of imaging quality.

Method	Target	Range IRW/cm	Range PSLR/dB	Range ISLR/dB	Azimuth IRW/cm	Azimuth PSLR/dB	Azimuth ISLR/dB
No correction	P_1_	8.03	−12.09	−7.83	7.93	−8.10	2.96
	P_5_	7.84	−12.74	−8.06	8.72	−7.83	0.96
Pitch angle correction	P_1_	7.66	−10.31	−5.80	7.53	−9.25	0.06
	P_5_	7.47	−10.96	−7.08	7.53	−8.10	0.50
Yaw angle correction	P_1_	7.66	−12.96	−10.07	7.93	−14.70	−12.00
	P_5_	7.66	−12.94	−10.03	7.93	−14.18	−12.03
All angle correction	P_1_	7.66	−12.96	−10.01	7.93	−15.95	−14.12
	P_5_	7.66	−12.97	−10.01	7.93	−15.78	−13.62

## References

[1] Marx D., Nelson M., Chang E., Gillespie W., Putney A., Warman K. An introduction to synthetic aperture sonar. Proceedings of the Tenth IEEE Workshop on Statistical Signal and Array Processing.

[2] Hansen R.E., Callow H.J., Saeboe T.O., Synnes S.A.V. (2011). Challenges in Seafloor Imaging and Mapping with Synthetic Aperture Sonar. IEEE Trans. Geosci. Remote Sens..

[3] Hayes M.P., Gough P.T. (2009). Synthetic Aperture Sonar: A Review of Current Status. IEEE J. Ocean. Eng..

[4] Griffiths H.D. Synthetic aperture imaging with sonar and radar: A comparison. Proceedings of the World Congress on Ultrasonics.

[5] Hayes M.P., Gough P.T. (2008). Interferometric synthetic aperture processing: A comparison of sonar and radar. J. Acoust. Soc. Am..

[6] Putney A., Chang E., Chatham R., Marx D., Nelson M., Warman L.K. Synthetic aperture sonar-the modern method of underwater remote sensing. Proceedings of the IEEE Aerospace Conference.

[7] Pengfei Z., Zelin J., Wei L., Jiyuan L., Chunhua Z. (2016). A motion compensation method for wide-swath synthetic aperture sonar. Chin. J. Acoust..

[8] Heremans R., Dupont Y., Acheroy M., Sergio S. (2009). Motion Compensation in High Resolution Synthetic Aperture Sonar (SAS) Images. Advances in Sonar Technology.

[9] Legris M., Jean F. Comparison between DPCA Algorithm and Inertial Navigation on the Ixsea Shadows SAS. Proceedings of the OCEANS 2007.

[10] Wilkinson D.R. (2001). Efficient Image Reconstruction Techniques for a Multiple-Receiver Synthetic Aperture Sonar. Ph.D. Thesis.

[11] Bellettini A., Pinto M. (2009). Design and Experimental Results of a 300-kHz Synthetic Aperture Sonar Optimized for Shallow-Water Operations. IEEE J. Ocean. Eng..

[12] Callow H.J., Hayes M.P., Gough P.T. (2009). Motion-Compensation Improvement for Widebeam, Multiple-Receiver SAS Systems. IEEE J. Ocean. Eng..

[13] Bonifant W.W., Richards M.A., McClellan J.H. (2000). Interferometric height estimation of the seafloor via synthetic aperture sonar in the presence of motion errors. IEE Proc.-Radar Sonar Navig..

[14] Gough P.T., Miller M.A. (2004). Displaced ping imaging autofocus for a multi-hydrophone SAS. IEE Proc.-Radar Sonar Navig..

[15] Leier S., Zoubir A.M. Quality assessment of synthetic aperture sonar images based on a single ping reference. Proceedings of the OCEANS 2007.

[16] Bellettini A., Pinto M.A. (2002). Theoretical accuracy of synthetic aperture sonar micronavigation using a displaced phase-center antenna. IEEE J. Ocean. Eng..

[17] Callow H.J. (2003). Signal Processing for Synthetic Aperture Sonar Image Enhancement. Ph.D. Thesis.

[18] Heremans R., Acheroy M., Dupont Y. 3B-6 Motion Compensation on Synthetic Aperture Sonar Images. Proceedings of the 2006 IEEE Ultrasonics Symposium.

[19] Hawkins D.W. (1996). Synthetic Aperture Imaging Algorithms: With application to wide bandwidth sonar. Ph.D. Thesis.

[20] Gough P.T., Hawkins D.W. (1997). Imaging algorithms for a strip-map synthetic aperture sonar: Minimizing the effects of aperture errors and aperture undersampling. IEEE J. Ocean. Eng..

[21] Banks S., Griffiths H. Imaging and motion estimation for synthetic aperture sonar based on fast factorised back-projection. Proceedings of the Sixth European Conference on Underwater Acoustics.

[22] Cumming I.G., Wong F.H. (2005). Digital Processing of Synthetic Aperture Radar Data: Algorithms and Implementation.

[23] Huang J., Tang J., Wang Q., Wu W. Motion Compensation in SAS with Multiple Receivers Based on ISCFT Imaging Algorithm. Proceedings of the 2nd International Conference on Information Engineering and Computer Science.

[24] Wu H., Tang J., Zhong H. A nonlinear chirp scaling algorithm for processing multi-receiver SAS data with small squint angle. Proceedings of the 2017 International Conference on Wireless Communication and Computer Science.

[25] Tian Z., Tang J., Zhong H., Zhang S. (2016). Extended Range Doppler Algorithm for Multiple-Receiver Synthetic Aperture Sonar Based on Exact Analytical Two-Dimensional Spectrum. IEEE J. Ocean. Eng..

[26] Bonifant W.W. (1999). Interferometic Synthetic Aperture Sonar Processing. Ph.D. Thesis.

[27] Zhang X., Tang J., Zhong H. (2014). Multireceiver Correction for the Chirp Scaling Algorithm in Synthetic Aperture Sonar. IEEE J. Ocean. Eng..

[28] Gebert N. (2009). Multi-Channel Azimuth Processing for High-Resolution Wide-Swath SAR Imaging.

[29] Hailiang Y. (2009). Studies on Imaging Algorithm of Multiple-Receiver Synthetic Aperture Sonar. Ph.D. Thesis.

